# Gold Nanorods as Radiopharmaceutical Carriers: Preparation and Preliminary Radiobiological In Vitro Tests

**DOI:** 10.3390/nano13131898

**Published:** 2023-06-21

**Authors:** Ludovica Binelli, Valentina Dini, Simone Amatori, Teresa Scotognella, Alessandro Giordano, Barbara De Berardis, Federica Bertelà, Chiara Battocchio, Giovanna Iucci, Ilaria Fratoddi, Antonella Cartoni, Iole Venditti

**Affiliations:** 1 Sciences Department, Roma Tre University, 00146 Rome, Italy; ludovica.binelli@uniroma3.it (L.B.); simone.amatori@uniroma3.it (S.A.); federica.bertela@uniroma3.it (F.B.); chiara.battocchio@uniroma3.it (C.B.); giovanna.iucci@uniroma3.it (G.I.); 2 Istituto Nazionale di Fisica Nucleare (INFN), Sezione di Roma3, Department of Sciences, Roma Tre University, 00146 Rome, Italy; 3 National Center for Innovative Technologies in Public Health, Istituto Superiore di Sanità, 00161 Rome, Italy; barbara.deberardis@iss.it; 4 Istituto Nazionale di Fisica Nucleare (INFN), Sezione di Roma1, Department of Physics, University La Sapienza, 00185 Rome, Italy; 5 Nuclear Medicine Unit, Fondazione Policlinico Universitario A. Gemelli IRCCS, 00168 Rome, Italy; teresa.scotognella@policlinicogemelli.it (T.S.); alessandro.giordano@unicatt.it (A.G.); 6 Department of Radiological and Hematological Sciences, Università Cattolica del Sacro Cuore, 00168 Rome, Italy; 7 Chemistry Department, Sapienza University, 00185 Rome, Italy; ilaria.fratoddi@uniroma1.it (I.F.); antonella.cartoni@uniroma1.it (A.C.)

**Keywords:** gold nanorods, technetium-99m, radiopharmaceuticals, theragnostic, nuclear medicine

## Abstract

Low-energy electrons (Auger electrons) can be produced via the interaction of photons with gold atoms in gold nanorods (AuNRs). These electrons are similar to those emitted during the decay of technetium-99m (^99m^Tc), a radioactive nuclide widely used for diagnostics in nuclear medicine. Auger and internal conversion (IC) electron emitters appropriately targeted to the DNA of tumors cells may, therefore, represent a new radiotherapeutic approach. ^99m^Tc radiopharmaceuticals, which are used for diagnosis, could indeed be used in theragnostic fields when loaded on AuNRs and delivered to a tumor site. This work aims to provide a proof of concept (i) to evaluate AuNRs as carriers of ^99m^Tc-based radiopharmaceuticals, and (ii) to evaluate the efficacy of Auger electrons emitted by photon-irradiated AuNRs in inducing radio-induced damage in T98G cells, thus mimicking the effect of Auger electrons emitted during the decay of ^99m^Tc used in clinical settings. Data are presented on AuNRs’ chemical characterization (with an aspect ratio of 3.2 and Surface Plasmon Resonance bands at 520 and 680 nm) and the loading of pharmaceuticals (after ^99m^Tc decay) on their surface. Spectroscopic characterizations, such as UV-Vis and synchrotron radiation-induced X-ray photoelectron (SR-XPS) spectroscopies, were performed to investigate the drug–AuNR interaction. Finally, preliminary radiobiological data on cell killing with AuNRs are presented.

## 1. Introduction

In the last decade, nanomaterials have found success in several fields, such as cosmetics, textiles, sensors, optoelectronics and medicine [1,2,3,4]. In the field of medicine, gold nanoparticles and gold nanorods (AuNRs) have found wide applications due to their peculiar chemical and physical features, such as the Localized Surface Plasmon Resonance (LSPR), which occurs when there is an interaction between a nanomaterial and electromagnetic radiation of the appropriate wavelength [5,6]. Another important outlook, which has favored the use of gold-based nanoparticles in biomedical applications, is their high biocompatibility. In fact, it is well known in the literature that gold nanoparticles are stable and biocompatible, even if these general characteristics also depend a lot on the specific dimension, shape and surface functionalization, and are studied on a case-by-case basis [7,8,9]. In particular, cetyltrimethylammonium bromide (CTAB)-functionalized AuNRs show different degrees of toxicity based on the types of performed tests. For example, Cornovale et al. found different cell viability for PC-3 cells (human prostate cancer cells) in free serum and supplemented serum, obtaining a range of 120–80% in viability using particle concentrations of 0.1–0.5 µg/mL in the substrate, respectively [10]. Guo et al. studied CTAB-functionalized gold nanoparticles’ cytotoxicity using the MTT test in a range of human and murine cells, and their study evidenced different results: in the PC-3 cell line, GR5, GR7, GR8, GR9, GR11 and G12 samples displayed IC50 values of 8.2, 7.8, 8.2, 7.4, 8.5 and 8.0 µg/mL, respectively. In contrast, Au NP-CTAB samples (20 and 60 nm) caused a higher level of cytotoxicity with IC50 values of 2 and 3.5 µg/mL, respectively [11]. The general low cytotoxicity of AuNRs has largely allowed their implementation in the diagnostic field, using near-infrared (NIR) imaging, due to their two plasmonic peaks that are associated with different electron oscillations on the transverse and longitudinal side of the rod in an energy range in which biological tissues are not active [12,13,14]. Many techniques exploit AuNRs in diagnosis, one of which is two-photon luminescence imaging (TPL), which is capable of going deep and has sub-micron resolution. To go even deeper into a tissue, photoacoustic tomography (PAT) can be used [15]. The underlying principle of PAT is the ability of AuNRs to absorb a pulsed laser and emit an acoustic shock wave due to transient superheating and thermoelastic expansion. Other techniques that have been developed using AuNRs are optical coherence tomography (OCT), where AuNRs are used to enhance contrast, and X-ray computed tomography (XCT), where they are used as contrast agents instead of iodine molecules [16]. It is also well known that AuNRs are used for drug delivery due to their high surface/volume ratio: the surface can be functionalized for optimizing the interaction with drugs in view of the specific targets and the therapy used [17]. One of the main objectives is the fight against tumors. In these cases, it is possible to exploit the Enhanced Permeability and Retention (EPR) effect, a passive targeting, or create active targeting systems using surface functionalizations of AuNRs with ligands that increase their specificity to target cells [18].

In this context, the field of nuclear medicine is studying with interest the use of drug delivery systems based on radioisotopes that emit short-range charged particles, instead of common drugs; these radioisotopes could be an added value to common used therapy because they are able to deliver a therapeutic dose of ionizing radiation to a tumor, causing cellular damage due to their interaction with biological macromolecules [19]. Increasing the dose delivered to a tumor mass while simultaneously decreasing the dose delivered to healthy tissues is still a major challenge in radiotherapy, although several strategies have been proposed. Furthermore, if the radioisotope used is also a gamma emitter, it can be used in medical diagnostic procedures (e.g., scintigraphy), and in this way, it is possible to have a theragnostic system that improves personalized therapy [20].

Several new radiopharmaceuticals have been prepared in recent years due to the discovery of kits called “*shake and bake*”, which are very easy to use and optimize to ensure that the desired complex has a high labeling yield and stability. Indeed, these new drugs are influenced by several factors: the amount of reducing agent and ligand, pH and temperature. In addition, since the beginning of the 21st century, there has been significant growing interest in the field of so-called nanomedicine with the use of nanomaterials labeled with radionuclides and used for both diagnostic and therapeutic purposes [21].

Among different radionuclides, technetium 99m (^99m^Tc), a gamma emitter, is widely used for diagnostic purposes in nuclear medicine, and several complexes labeled with it are available in clinical practice [22]. ^99m^Tc, during its nuclear decay, also emits Auger electrons (AEs), most of which has a low energy (<25 keV) and can traverse tissues for very short distances on the order of a few micrometers, resulting in a high Linear Energy Transfer (LET) between 1 and 23 keV/μm, which is very effective for producing clustered damage in the DNA and/or sensitive targets (e.g., cell membrane) of cancer cells. These types of damage are difficult to repair and generally lead to cell death [23]. The high lethality of AEs emitted near the nucleus is evident when observing their Relative Biological Effectiveness (RBE, defined as the ratio of the effectiveness of the radiation under investigation compared to X-rays or gamma rays used as the reference radiation); for AE radionuclides emitted from ^99m^Tc in rat thyroid PC Cl3 cells, and assuming a cellular or nuclear target for dose calculation, the RBE increased from 0.75 to 2.18 [24]. Therefore, AEs emitted from ^99m^Tc nuclear decay at the cellular level lead to a dense deposition of ionizing energy that is associated with increased radiobiological efficiency. If they are appropriately targeted to the DNA of tumor cells, they may represent an interesting new radiotherapy system: ^99m^Tc loaded AuNRs, delivered to the tumor site, colud indeed be used as a theragnostic radiopharmaceutical [25,26]. Similar to those emitted by the decay of ^99m^Tc, low-energy electrons (i.e., Auger electrons) are also produced by the interaction of photons with gold in AuNRs at energies below 1 MeV [27], thus providing a possible synergistic therapeutic effect on tumors if the therapeutic system is used in combination with conventional radiotherapy.

In this work, AuNRs were chosen as the drug delivery system (DDS) by exploiting their ease of synthesis and the possibility of subsequent surface engineering, as well as the presence of plasmonic absorption. This work is intended to be a proof of concept (i) to evaluate these new synthesized AuNRs as carriers of radiopharmaceuticals based on ^99m^Tc, and (ii) to evaluate the effectiveness of Auger electrons emitted by photon-irradiated AuNRs in inducing radio-induced damage at the cellular level, thus mimicking the effect of Auger electrons emitted during the decay of ^99m^Tc used in clinical settings. Preliminary data are presented on the chemical characterization of AuNRs (with typical Surface Plasmon Resonance bands in the visible range and an aspect ratio (A.R.) = 3.2) and the loading of radiopharmaceuticals based on long-lived ^99^Tc. Working with the decayed radiopharmaceutical has allowed us, on the one hand, to work with a compound already in use and with all the excipients present in the commercial compound, while, on the other hand, not having the safety limits imposed by a radioactive compound, thereby optimizing the chemical characterization and loading studies on AuNRs. To study the drug–AuNR interaction, spectroscopic characterizations, such as UV-Vis, Fourier-transform infrared (FTIR) and synchrotron radiation-induced X-ray photoelectron (SR-XPS) spectroscopies were performed. Finally, preliminary radiobiological data on cell killing with AuNRs are presented.

## 2. Materials and Methods

### 2.1. Materials for AuNR Synthesis and Conjugation

Cetyltrimethylammonium bromide (CTAB) (C_19_H_42_BrN, ≥97% Merck, Rahway, NJ, USA), tetrachoroauric (III) acid trihydrate (HAuCl_4_·3H_2_O, ≥99.9% Sigma-Aldrich, St. Louis, MO, USA), sodium borohydrate (NaBH_4_, 99.99% Aldrich, St. Louis, MO, USA), silver nitrate (AgNO_3_ 99.9%, Aldrich), L-ascorbic acid (C_6_H_8_O_6_, AA, 99% Sigma, St. Louis, MO, USA) and bidistilled H_2_O were used as received.

**Scheme 1:**
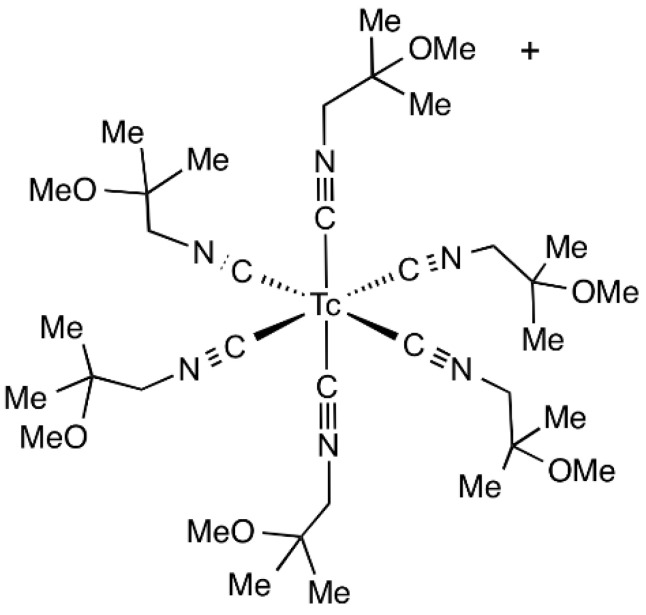
Chemical structure of ^99m^Tc-sestaMIBI.

^99m^Tc-sestaMIBI (chemical structure reported in Scheme 1) was chosen as the radiopharmaceutical: it is a methoxyisobutylisonitrile (MIBI) with an isonitrile group which together form a complex with ^99m^Tc. The labeling procedure was performed according to the manufacturer’s instructions (STAMICIS^®^, Curium Pharma, London, UK). The labeling procedure required the reconstitution of the vial with 3 mL (11.1 GBq) of fresh ^99m^TcO4, which was eluted from a ^99^Mo/^99m^Tc generator (Ultratechnekow FW, Curium Pharma). The vial was heated to 100 °C and incubated at room temperature for 15 min. The percentage of radiochemical purity (%RP) was assessed using an aluminum oxide strip (Agilent Technologies, Santa Clara, CA, USA) and ethanol as the eluent system, and it was analyzed via autoradiochromatography (Cyclone Plus^®^, Perkin Elmer, Waltham, MA, USA). The OptiQuant^®^ image analysis software was used to evaluate the %RP. After the quality control, ^99m^Tc-sestaMIBI was stored at 4 °C until completed decay. After the decay, long-lived ^99^Tc-sestaMIBI was used for loading on AuNRs.

### 2.2. Characterizations

The UV-Vis spectra were acquired in H_2_O by using a quartz cell with a Shimadzu 2401 PC UV-Vis spectrophotometer in a wavelength range between 200 and 800 nm. The Energy-Dispersive X-ray Analysis (FESEM_EDX) images were acquired using a MIRA3 Tescan instrument (resolution 200 nm, SEM HV 30.0 kV). Nanoparticles dispersed in Milli-Q water and in the cell culture medium RPMI1640 at the final concentration of 0.1 mg/mL were characterized by using a Zetasizer Ultra instrument (Malvern Instrument, Malvern, UK), in order to determine the hydrodynamic diameter (Z-Average) and the polydispersity index (PDI). The equilibration step at 25 °C was set for 2 min. Three determinations were performed based on 1 mL of sample suspensions. The values of Z-Average and PDI were determined using the ZS Xplorer Software (Malvern Instruments, UK). AuNR sample stability and surface charge were assessed based on Zeta-potential measurements. The measurements were conducted in triplicate with 750 μL of suspensions using an automatic measurement protocol of Zetasizer Ultra. A Mini Spin Eppendorf centrifuge was used for the purification of the AuNR samples (13,000 rpm, 15 min, and two times with bidistilled water). The high-resolution X-ray Photoelectron Spectroscopy (SR-XPS) measurements were performed in situ using the SuperESCA beamline of the Elettra synchrotron radiation facility in Trieste, Italy. The experimental chamber is equipped with a 150 mm Phoibos hemispherical electron energy analyzer (SPECS GmbH), provided with a homemade delay line detector. The high-resolution core level spectra were measured in the normal emission configuration while keeping the sample at RT. C1s and N1s core levels were recorded at 550 eV of photon energy while Tc3d and Au4f were measured at 300 eV in order to maximize the intensity of signals. The overall resolution was always better than 100 meV. For each spectrum, the binding energy scale was calibrated using the aliphatic C1s component (285.00 eV) as an internal reference.

### 2.3. AuNR Synthesis

For AuNRs, a two steps synthesis was used, in analogy with the literature [18]. The first step was to prepare the seed solution according to the following protocol. In a reaction flask, 5 mL of CTAB at 0.2 M and 5 mL of HAuCl_4_ at 0.0005 M were added. The solution was stirred and degassed with Argon for 3 min, and then 600 μL of NaBH_4_ at 0.01 M was added. At this point, a color change was observed (the solution assumed the typical brownish-yellow coloration), and the solution was left in agitation for 5 min. In the second step, the growth solution was added 10 mL of CTAB at 0.2 M, 10 mL of HAuCl_4_ at 0.001 M, and 400 μL of AgNO_3_ at 0.004 M. The solution was stirred and degassed using Argon for 3 min, and then 70 μL of AA at 0.078 M and 24 μL of the seed solution were added. The solution was left in agitation for 20 min. For purification, the suspension was centrifugated at 13,000 rpm for 15 min, for two times. The AuNRs that were used for the biological test were synthesized following the experimental conditions shown in Table 1.

### 2.4. Preparation of Conjugate Nanorods

To evaluate the loading of radiopharmaceutical on AuNRs, two tests were carried out, which were always performed in triplicate, following the protocol reported here. A total of 4 mL of the long-lived ^99^Tc-sestaMIBI solution (0.0165 mg/mL) was placed in three vials with 1 mL of the AuNR solution (1 mg/mL). The solution was left in agitation for 24 h and, at the end, was centrifugated at 13,000 rpm for 20 min. The conjugate was stored at −20 °C, while the supernatant was used for the loading evaluation. From now on, the decayed, non-radioactive radiopharmaceutical will be indicated with ^99^Tc and its conjugate on gold rods with AuNRs-99Tc.

### 2.5. Cell Culture

Human glioblastoma multiform cells (T98G cells) were purchased from the European Collection of Authenticated Cell Cultures (ECACC, UK Health Security Agency). The cells were grown in monolayer at 37 °C in a humidified atmosphere of 95% air and 5% CO_2_, in RPMI-1640 medium (Euroclone S.p.A., Pero, Italy) supplemented with 10% fetal bovine serum (GIBCO^®^, Life Technologies, Waltham, MA, USA), l mM of glutamine (Euroclone S.p.A., Italy), and 50 U/dm^3^ of penicillin and streptomycin (Euroclone S.p.A., Italy) with a doubling time of 27 h.

The flasks containing asynchronous non-confluent cells were gently rinsed with 10 mL of calcium and magnesium-free D-PBS (GIBCO^®^, Life Technologies, Waltham, MA, USA), and then detached using 1 mL of 1:1 *v/v* solution of 0.25% trypsin and 1 × 10^−3^ M EDTA. Trypsin was neutralized using a few mL of the fresh culture medium, and the cell solution was counted (using a Coulter Counter Z2 serie, Beckmann, Kristiansand, Norway). The T98G cells employed for the experiments were seeded on T-25 flasks at a concentration of 8 × 10^3^ cells/cm^2^ at 48 h before the experiment. At 24 h before the experiment, the cell culture medium was removed and replaced with a fresh medium or a medium containing AuNRs, at two different concentrations (0.1 μg/mL and 0.5 μg/mL), and placed in the incubator. On the day of the experiment, the cell culture medium was removed, and every flask was replaced with the fresh medium 1 h before irradiation. Some of the flasks were irradiated with doses of 1 Gy and 4 Gy. Then, the cells were detached from all flasks, counted and, through a series of successive dilutions, seeded in appropriate numbers in 4 Petri dishes at a final volume of 5 mL, as shown in Table 2.

### 2.6. Irradiation

Irradiation with gamma rays (E = 0.662 MeV) was performed at the Istituto Superiore di Sanita’ (ISS, Rome, Italy) with doses of 1 Gy and 4 Gy at a dose rate of 0.6 Gy/min using a ^137^Cs source (Gammacell 40, Nordion Inc., Ottawa, ON, Canada). The doses were selected based on previous data [24]. All irradiations were performed at room temperature.

### 2.7. Colony-Forming Assay

To study the cytotoxic effect of AuNRs on T98G cells (in terms of reproductive cell death), the colony-forming assay developed by Puck and Marcus was used [28]. The assay allows the assessment of classical clonogenic survival. Briefly, after the treatment (i.e., with pristine AuNRs, upon γ-ray irradiation only and in combination), the cells were trypsinized, counted and plated into four 6 cm Petri dishes at the appropriate concentration to score the number of colonies ranging from 300 to 600 for each dose, as shown in Table 2. After about 12 days of growth at 37 °C under 5% CO_2_ and 95% humidity, the colonies were stained with crystal violet (Figure S1 in Supplementary Materials). Colonies exceeding 50 cells were scored manually and represented surviving cells. The average colony count for the four Petri dishes was used to calculate plating efficiency (PE), which was defined as the number of colonies counted/number of cells plated. For each experiment, cell-surviving fractions (SF) were calculated as the ratio between the PE measured in the investigated sample and the measured PE of the corresponding control. All significance was calculated using Student’s-*t*-test.

## 3. Results

### 3.1. AuNR Synthesis and Loading Studies

The choice of AuNRs as a drug carrier is strategic because it allows us to combine the high stability and biocompatibility of gold with the anisotropic form, which allows a versatile engineering possibility [6]. Furthermore, AuNRs possess specific plasmonic properties, presenting two plasmonic peaks in the visible and/or near-infrared spectral range.

**Figure 1:**
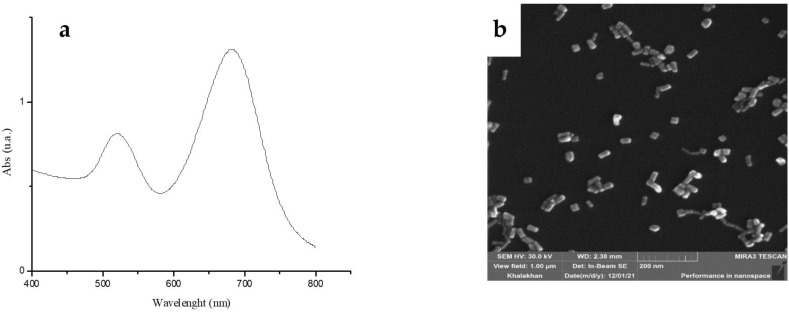
AuNRs: (**a**) visible spectrum in water suspension, with λ max = 520–680 nm, and (**b**) FESEM-EDX image (scale bar 200 nm), with rods having average dimensions of 39 ± 5 × 11 ± 2 nm (A.R. = 3.2).

In this work, AuNR synthesis was performed in accordance with recent literature [29] and consisted of two steps. In the first step, Au^3+^ was reduced by NaBH_4_ in the presence of CTAB, and a brownish-yellow solution, i.e., the seed solution, was obtained. In the second step, which concerned the growth of nanorods, the reduction of gold by ascorbic acid was initiated, and this allowed the conversion of gold from Au^3+^ to Au^1+^ and then to Au^0^. The AuNR spectrum in water (Figure 1a) shows the two typical plasmon bands at 520–680 nm, confirming the nanodimension of the material. FESEM EDX studies were performed, and average sizes of 39 ± 5 nm and 11 ± 2 nm and the respective A.R. = 3.2 were observed (Figure 1b).

Moreover, DLS studies were performed in water and in the cell culture medium RPMI1640. These data, presented in Table S1 in Supplementary Materials, show high values of PDI mainly due to the non-spherical shape of the particles, which leads to its incorrect determination and an overestimation of the hydrodynamic diameter. The differences observed between the AuNR characterization data obtained via the DLS and SEM analyses are related to the shape of nanoparticles and the different physical phenomena used to determine size distribution. SEM measures the true diameter, while DLS measures hydrodynamic diameter, leading to misleading results for non-spherical particles [30]. Both AuNRs and AuNRs-99Tc showed negative surface charge, and when AuNRs were suspended in the cell culture medium, the Zeta-potential values increased, suggesting adsorption of the protein corona. The AuNRs’ stability was checked over time using UV-vis and DLS studies up to one month after their synthesis, confirming their dimension and polydispersity. For evaluating drug loading on the AuNR surface, a calibration curve was performed (y = 55.409x and R^2^ = 0.999, as reported in Figure S2 in Supplementary Materials) using different concentrations of the drug [31]. The protocol for loading ^99^Tc-sestaMIBI onto the rods’ surface was investigated via the simple contact between the radiopharmaceutical solution and the suspension of AuNRs for 24 h under stirring at room temperature. The obtained loading efficiency was η (%) = 5 ± 2%, which corresponds to 0.0033 mg of the drug for 1 mg of AuNRs.

### 3.2. Synchrotron Radiation-Induced X-ray Photoelectron Spectral (SR-XPS) Studies

**Figure 2:**
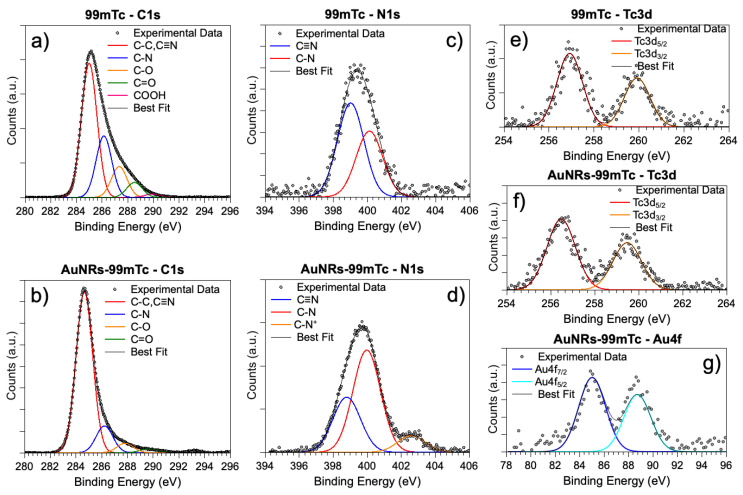
SR-XPS spectra: C1s (**a**,**b**), N1s (**c**,**d**) and Tc3d (**e**,**f**) of decayed and non-radioactive radiopharmaceutical ^99^Tc and its conjugate on gold rods, AuNRs-99Tc. SR-XPS Au4f spectrum of AuNRs-99Tc (**g**).

SR-XPS measurements were performed on AuNRs-99Tc in order to ascertain the successful loading of the radiopharmaceutical onto AuNRs and to obtain a better insight into the stability of the ^99^Tc molecular structure upon conjugation to the surface of AuNRs. For comparison, SR-XPS measurements were also carried out on ^99^Tc. The SR-XPS spectra were acquired at the C1s, N1s and Tc3d core levels and, for the ^99^Tc-sestaMIBI-AuNRs sample, at the Au4f core level as well. Complete SR-XPS data analysis results (binding energy (BE), full width-half maximum (FWHM), and atomic percentages and proposed assignments for all measured signal components) are summarized in Table S2 in Supplementary Materials. All signals appeared composite, and by applying a peak fitting procedure, several spectral components were individuated and assigned, based on a comparison with the literature [32], to the specific elements in the respective chemical groups. As shown in Figure 2, the measured spectra for the C1s and N1s core levels confirm the stability of the ^99^Tc molecular structure, and for the AuNRs-99Tc system, the presence of CTAB, as expected by the nanorods’ chemical composition.

In more detail, the intensity increment observed for the C1s component at 285.0 eV of BE (C-C) in AuNRs-99Tc, with respect to the analogous signal in pristine 99Tc, is associated with the aliphatic tail of CTAB (Figure 2a,b); an analogous effect is observed for the N1s spectral features associated with amine-like N (Figure 2c,d) with respect to the peak component assigned to the C≡N groups. On the other hand, the intensity of the third peak in the C1s spectra (287.3 eV for BE), arising due to the C-O groups of the -OMe moieties of ^99^Tc, decreases in the AuNRs-99Tc sample, as expected. The C1s peaks at higher B.E. assigned to C=O (about 288 eV) and COOH (290 eV) reveal the presence of impurities, which are always observed in the samples prepared in air via deposition from aqueous solutions. To further prove the effectiveness of the ^99m^Tc-sestaMIBI conjugation to the AuNR surface, Au4f and Tc3d signals were analyzed. For Au4f (Figure 2g), a single spin–orbit pair is observed, with the main Au4f_7/2_ component being centered at 85.04 eV, as expected for gold atoms at the AuNRs surface interacting with ligands [33]. Finally, the reproducibility of Tc3d spectral position and shape [34,35] fully confirms the effectiveness of the loading process (Figure 2e,f).

### 3.3. Biological Studies

**Figure 3:**
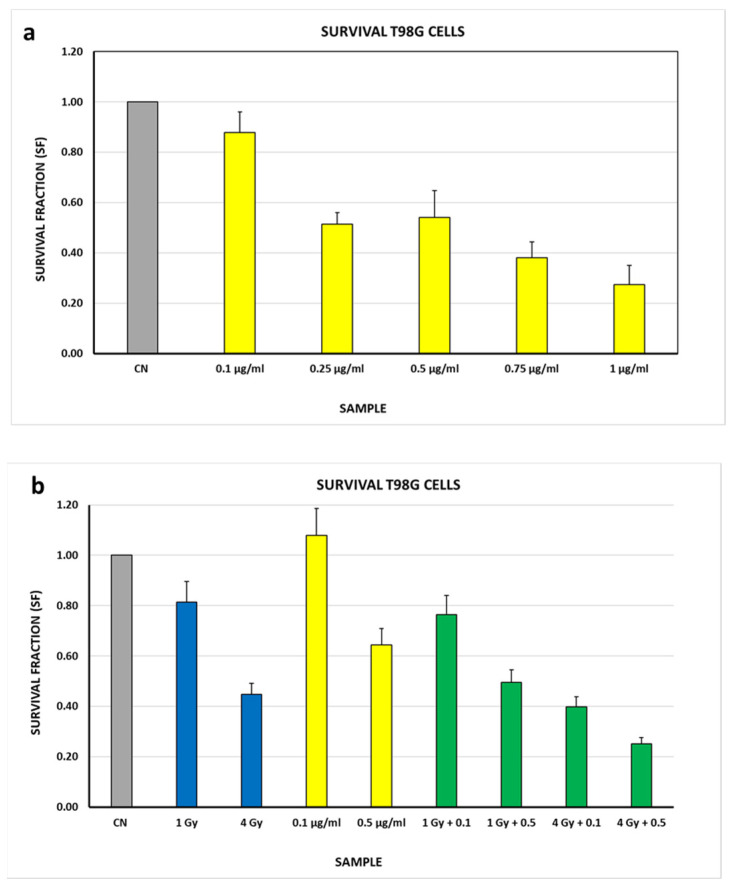
Bar plot for the results of the SF obtained from the colony-forming assay in glioblastoma T98G cells. Panel (**a**) shows the SF after treatment with different AuNR concentrations (yellow histograms). Panel (**b**) shows the SF after different treatments: blue histograms (only γ-rays), yellow histograms (only AuNRs), and green histograms (γ-rays + AuNrs). All results are significant compared to the control with a *p* value of <0.01, except for 0.1 μg/mL in panel (**b**). Significance was calculated using Student’s *t*-test. The error bar represents the standard error of the mean (SEM) obtained from at least 3 independent experiments for each condition used.

To evaluate the cytotoxic effect of AuNRs, alone or in combination with gamma ray (γ-ray) irradiation, the cellular reproductive death in T98G cells was studied using the colony-forming assay. Figure 3a shows that the SF of the samples treated only with AuNRs, in the concentration range of 0.1–1 µg/mL, decreases as the concentration increases, as expected based on a comparison with the literature [36,37,38,39]. For subsequent experiments with the combined treatment (i.e., AuNRs and γ-ray irradiation), we arbitrarily chose 0.1 µg/mL and 0.5 µg/mL because the former shows no toxicity whereas the latter reduces SF by approximately 50%. The results are shown in Figure 3b, along with the results of the samples treated with γ-rays only. In particular, the latter are consistent with the literature data [40,41,42,43].

In this analysis, all samples were normalized to the same control, (untreated cells—CN). Overall, it can be seen that the SF decreases as the dose and concentration of AuNRs increase (Figure 3b). In the samples with the combined treatment (i.e., AuNRs and γ-ray irradiation), the SF decreases at the same dose as the concentration of AuNRs increases, with an overall trend that appears to be linear. The decrease observed in the SF of the samples tested with the combined treatment can be due to three factors: the damage induced by γ-ray irradiation, the cytotoxicity of AuNRs, and the radiation damage due to the emission of Auger electrons from the irradiated gold.

**Figure 4:**
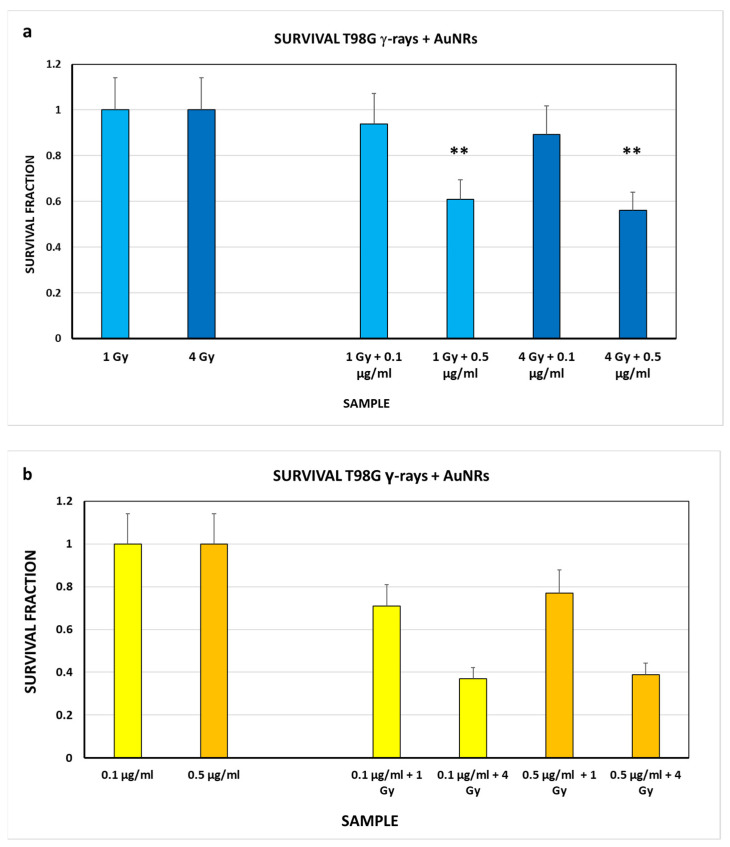
Bar plot of the SF obtained after normalizing to the dose used (**a**) or normalizing to the AuNR concentration (**b**). Significant difference with respect to the control was calculated using an ANOVA test (** *p* < 0.01). The error bar represents the propagation of the error.

To better understand which of the three factors most strongly affected cell survival in these samples, a more detailed analysis was conducted. The SF was calculated by considering the samples treated only with γ-rays as the control first (Figure 4a), and considering the samples treated only with AuNRs as the control afterward (Figure 4b).

In the former case (panel a), a similar decrease in the SF is observed for both doses as the AuNR concentration increases, but the decrease is significant with respect to the control only for the concentration of 0.5 μg/mL. The obtained data show that the observed effect is attributable to the presence of AuNRs and Auger electron emission from the irradiated gold.

In the latter case (panel b), the obtained data show that the SF decreases as the radiation dose increases, which is significant for all conditions with respect to the control. Furthermore, a significant decrease in SF is observed for the sample treated with 0.1 µg/mL or 0.5 µg/mL of AuNRs and irradiated at 4 Gy compared to the sample treated with 0.1 µg/mL or 0.5 µg/mL AuNRs and irradiated at 1 Gy. In this case, by normalizing the data to the AuNR concentrations used, the obtained results show that the decrease in SF is attributable to gamma rays and Auger electron emission from the irradiated gold.

The contribution of Auger electrons could be calculated as a first approximation by subtracting the SF values of the samples in panel b from the SF values of the samples treated with gamma rays only (see Figure 3b). The results are shown in Table 3.

The obtained results seem to indicate that emission of Auger electrons from the irradiated gold of AuNRs occurs, although the energy of incident photons is only slightly higher than the suitable energy, and under our experimental conditions, it seems to be dose dependent rather than AuNR concentration dependent.

## 4. Conclusions

In this study, AuNRs were synthesized (A.R. = 3.2), fully characterized and conjugated with long-lived ^99^Tc-sestaMIBI (STAMICIS^®^), a tumor-seeking radiopharmaceutical which is currently used in diagnostic imaging. ^99^Tc was used in the non-radioactive form after complete decay. The obtained AuNRs were studied as a proof of concept from a radiobiological point of view to obtain an overall view of theragnostic action. In fact, the presence of the radiopharmaceutical used in diagnosis could induce the emission of Auger electrons from AuNRs, which has a therapeutic effect at the diagnostic site. This effect was explored as a proof of concept through the irradiation of AuNRs with an external γ source. The cell killing tests were performed on T98G cells. It emerged that in the case of AuNRs alone without radiation, the lowest concentration of 0.1 μg/mL was not toxic for the cells, while a concentration of 0.5 μg/mL showed some degree of toxicity. For the samples treated with both irradiation and AuNRs, the trend of SF appears to be linear: as the radiation dose and AuNR concentration increase, cell survival decreases. The effect of the induced AE emission on cell survival does not appear to be predominant. In fact, from our preliminary data, it appears to be the dose of gamma rays with which the cells are irradiated that contributes most significantly and in an independent manner from the concentration of AuNRs used. Further studies are needed to optimize the loading of this radiopharmaceutical onto AuNRs, and further biological tests, with and without irradiation, will be necessary to evaluate the mechanism and efficiency of action. However, these preliminary investigations are fundamental to verify and optimize the loading of a decayed radiopharmaceutical, thereby minimizing the dangers for operators and the costs, and, above all, they allow the optimization of the protocols under consideration, by mimicking the action of radionuclide on gold, in view of the loading of the radioactive drug, which would lead to the realization of the theragnostic system.

## Figures and Tables

**Table 1:** Experimental conditions for the synthesis of AuNRs.

CTAB mL (M)	HAuCl_4_ mL (M)	AgNO_3_ μL (M)	AA μL (M)	Seed Solution, μL
10 (0.2)	10 (0.001)	400 (0.004)	70 (0.078)	24

**Table 2:** Experimental conditions utilized for the colony-forming assay.

Sample	Dose (Gy)	AuNRs (μg/mL)	Cells Plated
CN	-	-	400
0.1 μg/mL	-	0.1	400
0.5 μg/mL	-	0.5	400
1 Gy	1	-	400
4 Gy	4	-	1000
1a	1	0.1	900
2a	1	0.5	2000
3a	4	0.1	3000
4a	4	0.5	4000

Legend: CN: control, i.e., cells not treated; 0.1 μg/mL and 0.5 μg/mL: the AuNR concentration tested; 1 Gy and 4 Gy: the radiation dose used; 1a–4a: the combination treatment.

**Table 3:** Contributions of the various components normalized to the radiation dose used.

Sample	SF (see Figure 3b)	Sample	SF (see Figure 4b)	Effect of Auger Electrons
CN	1 ± 0.14	0.1 μg/mL 0.5 μg/mL	1 ± 0.14 1 ± 0.14	- -
1 Gy	0.81 ± 0.14	0.1 μg/mL + 1 Gy 0.5 μg/mL + 1 Gy	0.71 ± 0.10 0.77 ± 0.11	0.11 ± 0.13 0.05 ± 0.14
4 Gy	0.45 ± 0.14	0.1 μg/mL + 4 Gy 0.5 μg/mL + 4 Gy	0.37 ± 0.05 0.39 ± 0.05	0.08 ± 0.07 0.06 ± 0.07

## Data Availability

Data supporting are in Supporting Materials.

## Supplementary Materials

The following supporting information can be downloaded at https://www.mdpi.com/article/10.3390/nano13131898/s1. Figure S1: Photo images of Petri dishes containing the colonies, which have been stained with crystal violet and are representative of the respective biological samples being analyzed; Figure S2: Calibration curve for ^99^Tc-sestaMIBI in water (error bar showing the standard deviation is not appreciable); Table S1: Values of Z-Average, PDI and Zeta potential; Table S2: XPS data collected on the radiopharmaceutical before and after conjugation to AuNRs.
