# Supplementary material for: Gold Nanorods as Radiopharmaceutical Carriers: Preparation and Preliminary Radiobiological In Vitro Tests
Source: Nanomaterials (Basel). 2023 Jun 21;13(13):1898. doi: 10.3390/nano13131898 (PMC10343762; doi:10.3390/nano13131898)
Supplement: Supplementary file 1 [file nanomaterials-13-01898-s001.zip › nanomaterials-2442000-supplementary.pdf]

SUPPLEMENTARY MATERIALS

Gold nanorods as radiopharmaceuticals carriers: preparation and preliminary radiobiological in vitro tests

Ludovica Binelli[1,2, ¥], Valentina Dini[3,4,¥,*], Simone Amatori[1], Teresa Scotognella[5], Alessandro Giordano[5,6], Barbara De Berardis[3], Federica Bertelà[1], Chiara Battocchio[1], Giovanna Iucci[1], Ilaria Fratoddi[7], Antonella Cartoni[7], Iole Venditti[1*]

[1]Sciences Department, Roma Tre University, Rome; ludovica.binelli@uniroma3.it; simone.amatori@uniroma3.it; federica.bertela@uniroma3.it; chiara.battocchio@uniroma3.it; giovanna.iucci@uniroma3.it; iole.venditti@uniroma3.it;

[2]Istituto Nazionale di Fisica Nucleare (INFN), Sezione di Roma3, Department of Sciences Roma Tre University Rome, Italy

[3]National Center for Innovative Technologies in Public Health, Istituto Superiore di Sanità, 00161 Rome, Italy; valentina.dini@iss.it; barbara.deberardis@iss.it

[4] Istituto Nazionale di Fisica Nucleare (INFN), Sezione di Roma1, Department of Physics, University La Sapienza, 00185 Rome, Italy

[5] Nuclear Medicine Unit, Fondazione Policlinico Universitario A. Gemelli IRCCS, 00168 Rome, Italy; teresa.scotognella@policlinicogemelli.it; alessandro.giordano@unicatt.it

[6] Department of Radiological and Hematological Sciences, Università Cattolica del Sacro Cuore, 00168 Rome, Italy

[7]Chemistry Department Sapienza University 00185 Rome Italy; ilaria.fratoddi@uniroma1.it; antonella.cartoni@uniroma1.it

*Correspondence: iole.venditti@uniroma3.it, valentina.dini@iss.it

¥these Authors contribute equally

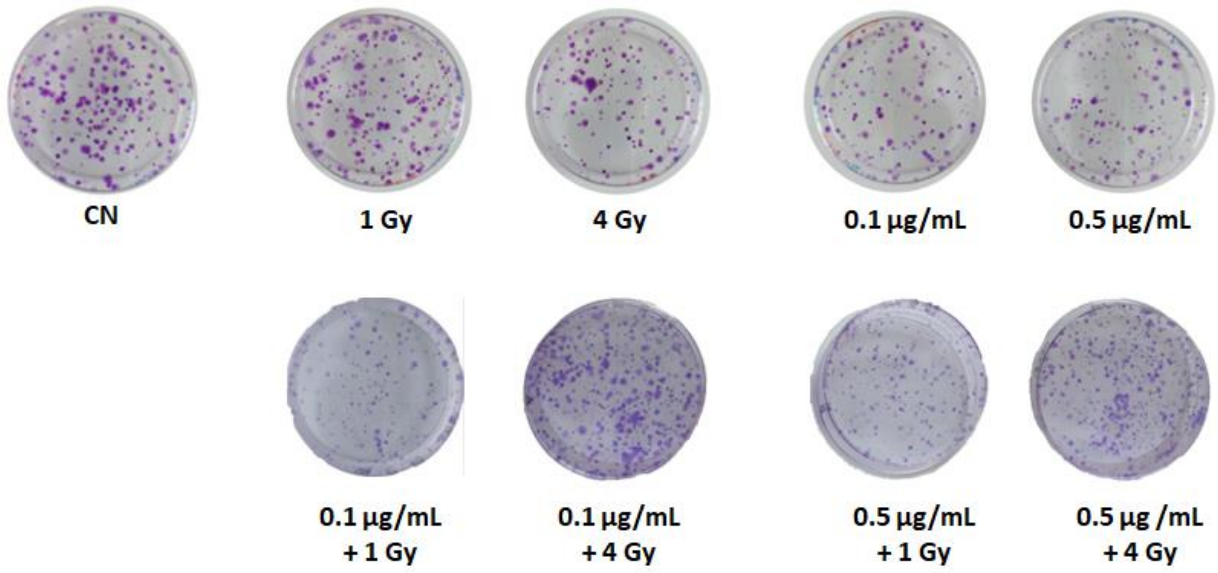

Figure S1. Photo images of the Petri dishes containing the colonies, stained with crystal violet and representative of the respective biological samples analysed.

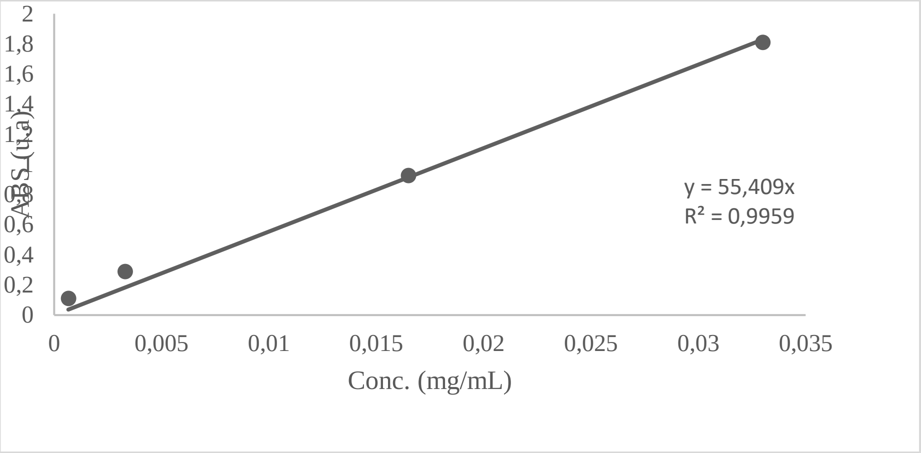

Figure S2. Calibration Curve of ^{99}Tc-sestaMIBI in water (error bar to quote standard deviation is not appreciable).

Table S1. Values of Z-Average, PDI and Zeta potential for AuNRs

Nanoparticles	Medium	Z-average (nm)	PDI	Zeta potential (mV)
AuNRs	Milli-Q water	98.2 ± 2.0	0.545 ± 0.038	-21.1 ± 0.4
AuNRs	RPMI	39.9 ± 30.8	0.697 ± 0.500	-11.8 ± 1.0
AuNRs-^{99}Tc	Milli-Q water	972.0± 188.0	0.994 ± 0.116	-10.8 ± 1.1
AuNRs-^{99}Tc	RPMI	42.9 ± 0.1	0.3360 ± 0.0316	-9.7 ±1.0

Table S2. XPS data collected on the radiopharmaceutical before and after conjugation to the AuNRs.

AuNRs-^{99}Tc	***B.E. (eV)***	***FWHM***	***Atomic Ratios***	***Assignment***
C1s	285.00<br>286.29<br>287.33<br>288.28<br>290.49	1.20<br>1.20<br>1.20<br>1.20<br>1.20	9.4<br>2.6<br>1.0<br>0.9<br>0.4	C-C, C≡N<br>C-N<br>C-O<br>C=O (impurities)<br>COOH (impurities)
Tc3d$_{5/2}$	257.09	1.53	1	Tc(I)
Au4f$_{7/2}$	85,04	2,59	1	AuNRs surface
N1s	398.79<br>399.97<br>402.60	1.91<br>1.91<br>1.91	1.0<br>1.8<br>0.3	C≡N<br>N-C<br>N^+-C
99Tc	***B.E. (eV)***	***FWHM***	***Atomic Ratios***	***Assignment***
C1s	285<br>286.14<br>287.34<br>288.53<br>289.93	1.43<br>1.43<br>1.43<br>1.43<br>1.43	4.3<br>2.0<br>1.0<br>0.5<br>0.1	C-C, C≡N<br>C-N<br>C-O<br>C=O (impurities)<br>COOH (impurities)
Tc3d$_{5/2}$	256.83	1.34	1	Tc(I)
N1s	399.01<br>400.12	1.83<br>1.83	1.0<br>0.7	C≡N<br>N-C
